# Acute Effects of *Salvia* Supplementation on Cognitive Function in Athletes During a Fatiguing Cycling Exercise: A Randomized Cross-Over, Placebo-Controlled, and Double-Blind Study

**DOI:** 10.3389/fnut.2021.771518

**Published:** 2021-11-30

**Authors:** Nicolas Babault, Ahmad Noureddine, Nicolas Amiez, Damien Guillemet, Carole Cometti

**Affiliations:** ^1^INSERM UMR1093-CAPS, Université Bourgogne Franche-Comté, UFR des Sciences du Sport, Dijon, France; ^2^Centre d'Expertise de la Performance, Université Bourgogne Franche-Comté, UFR des Sciences du Sport, Dijon, France; ^3^Nexira, Rouen, France

**Keywords:** memory, attention, fatigue, perceived exertion, supplementation, reaction time

## Abstract

**Background:** *Salvia* (sage) supplementation has been shown to improve the cognition function in healthy individuals or patients (e.g., attention, memory). To date, no study has explored its relevancy in the context of sporting performance. The aim of this study was to explore the acute effects of a combination of *Salvia officinalis* and *Salvia lavandulaefolia* on cognitive function in athletes performing a fatiguing cycling task.

**Methods:** Twenty-six volunteers were included in this cross-over, randomized, double-bind vs. placebo trial. Two hours before the two experimental sessions (here called SAGE and PLACEBO), volunteers randomly received a supplementation of sage or placebo. During each experimental session, participants were tested at four occasions while cycling during a warm-up, in the middle and at the end of a fatiguing task and after a short 5-min recovery. Tests included a Stroop task, a simple reaction time task, and a backward digit span memory task. Heart rate and rating of perceived exertion (RPE) were also measured at the beginning of the four test sessions.

**Results:** Heart rate was significantly greater during the fatiguing exercise than during warm-up and recovery (*P* < 0.001) without any supplementation effect. RPE was greater during the fatiguing exercise than during warm-up and recovery (*P* < 0.001). Moreover, RPE was significantly lower during the SAGE session as compared to PLACEBO (*P* = 0.002). Reaction time was not altered during the exercise but was significantly shorter with SAGE as compared to PLACEBO (*P* = 0.023). The Stroop task only revealed significantly longer reaction time during warm-up as compared to recovery (*P* = 0.02) independently of the supplementation. The digit span memory test revealed a significant greater span score with SAGE as compared to PLACEBO (*P* = 0.044).

**Conclusion:** The combination of *Salvia* improved the cognitive functions (perceived exertion, working memory, and reaction time). The positive effects were obtained in fresh condition and were maintained with fatigue.

## Introduction

Multiple nutritional strategies could be used to improve performance of competitive athletes. Long-term (chronic) diets may be beneficial for multiple physiological adaptations such as gains of muscle mass using proteins (1). Acute supplementations may also enhance numerous aspects of performance (2). Supplementation may act at different physiological levels, including the central nervous system. For instance, supplementation could reduce serotonin synthesis or increase the concentration of neurotransmitters and therefore lower mental fatigue or increase the cognitive function (i.e., attention or vigilance) (2, 3). Confirming this assumption, the authors have observed that the negative effects of fatigue during a long duration offshore sailing race (feeling of fatigue and decrease in memory) were lowered with protein feeding (4). Nutrition supplementation could therefore be beneficial during numerous sporting situations for physical performance (i.e., improve performance and delay fatigue) and/or for neural and cognitive performance (e.g., improve attention and vigilance for fast decision makings or short reaction times).

Numerous nutrients have been proposed to act at these different levels. Among the wide possibilities, *Salvia* species are promising candidates according to their various active constituents such as caffeic acid, salvianolic acid, sagecoumarin, and sagerinic acid (5). Among the possible effects (5) of *Salvia* have been demonstrated effective for acetylcholinesterase inhibition (6) that would likely originate from the monoterpenoid constituents (7). Concomitant improved mood and cognitive performance were observed (6, 7). Terpenoids (e.g., eucalyptol, camphor, α-, β-pinene, and others), particularly present in *Salvia* essential oil, have also been shown to include antioxidant effects by inhibiting reactive oxygen species production and increasing endogenous antioxidant compounds (8). Anti-inflammatory effects are also observed as a result of phenolic diterpenes present in some species of *Salvia* (e.g., *Salvia officinalis*) that reduce, for instance, cytokines or interleukins (5). Different trials have shown positive effects for attention, memory, speed of memory, alertness, or calmness after single intake in healthy individuals (9–11). *Salvia* also includes numerous polyphenols (e.g., rosmarinic acid, apigenin glucosides, luteolin glucosides, and others) components with a well-known antioxidant effect (5, 12). Polyphenols are of particular interest for endurance-type performance. Indeed, in a preceding study, we have observed the positive effects of acute apple and grape polyphenols intake to increase cycling time to exhaustion and delay the maximal perceived exertion (13).

*Salvia* plants include numerous species with considerable constituent variations and resultant potential effects (5). To date, most clinical studies have confirmed the acute and chronic effects of a given particular species. In contrast, a recent study demonstrated that a combination of *Salvia* was more efficient than a single constituent (14). This study observed larger improvements in memory and learning (Y-maze and Morris water maze tests) on *in vivo* rodents using a combination of *S. officinalis* (leaf extract mostly characterized for polyphenol content) and *Salvia lavandulaefolia* (encapsuled essential oil with predominant terpenoids) (14). This specific combination of *Salvia* has also been demonstrated efficient for working memory in healthy humans using various tasks (9). However, to date and to the best of our knowledge, no study has explored the effects of *Salvia*, in sports or physical activity settings. The aim of this study was to explore the acute effects of a supplementation of *S. officinalis* and *S. lavandulaefolia* on cognitive function in athletes performing a fatiguing cycling task. According to the positive effects of *Salvia* on cognitive function, we hypothesized that *Salvia* will improve cognition during exercise and that these positive effects were exacerbated in a fatigue state.

## Materials and Methods

### Study Design

This study was a cross-over, randomized, double-blind, and placebo-controlled trial. All volunteers came to the laboratory on four separate occasions (familiarization, initial testing, and two experimental sessions) with 7 days between each session. All experimental sessions were performed the same day of the week, at the same hour of the day (during the morning, 2 h after a standardized breakfast). During the total duration of the study, volunteers were instructed to refrain from intensive exercise.

The familiarization session aimed to (i) explain the experimental procedure, (ii) determine anthropometrics (age, height, body mass, and percentage of fat mass), and (iii) familiarize volunteers with the different tests at rest and while cycling at a light power output (ranging from 60 to 80 W with a minimum of 60 rpm, revolutions per minute). Body mass and percentage of fat mass were measured using a Tanita BC420 (Tanita, Tokyo, Japan). Cycling was performed on an air- and magnet-braked cycle ergometer Wattbike Pro (Wattbike Ltd., Nottingham, United Kingdom), with saddle and handlebar individually adjusted (settings were noted and used during the other sessions). Then, the initial testing session aimed to (i) determine the maximal aerobic power (MAP) using an incremental cycling test and (ii) re-familiarize volunteers with the different tests at rest and while cycling at a light power output (ranging from 60 to 80 W with a minimum of 60 rpm).

**Figure 1:**
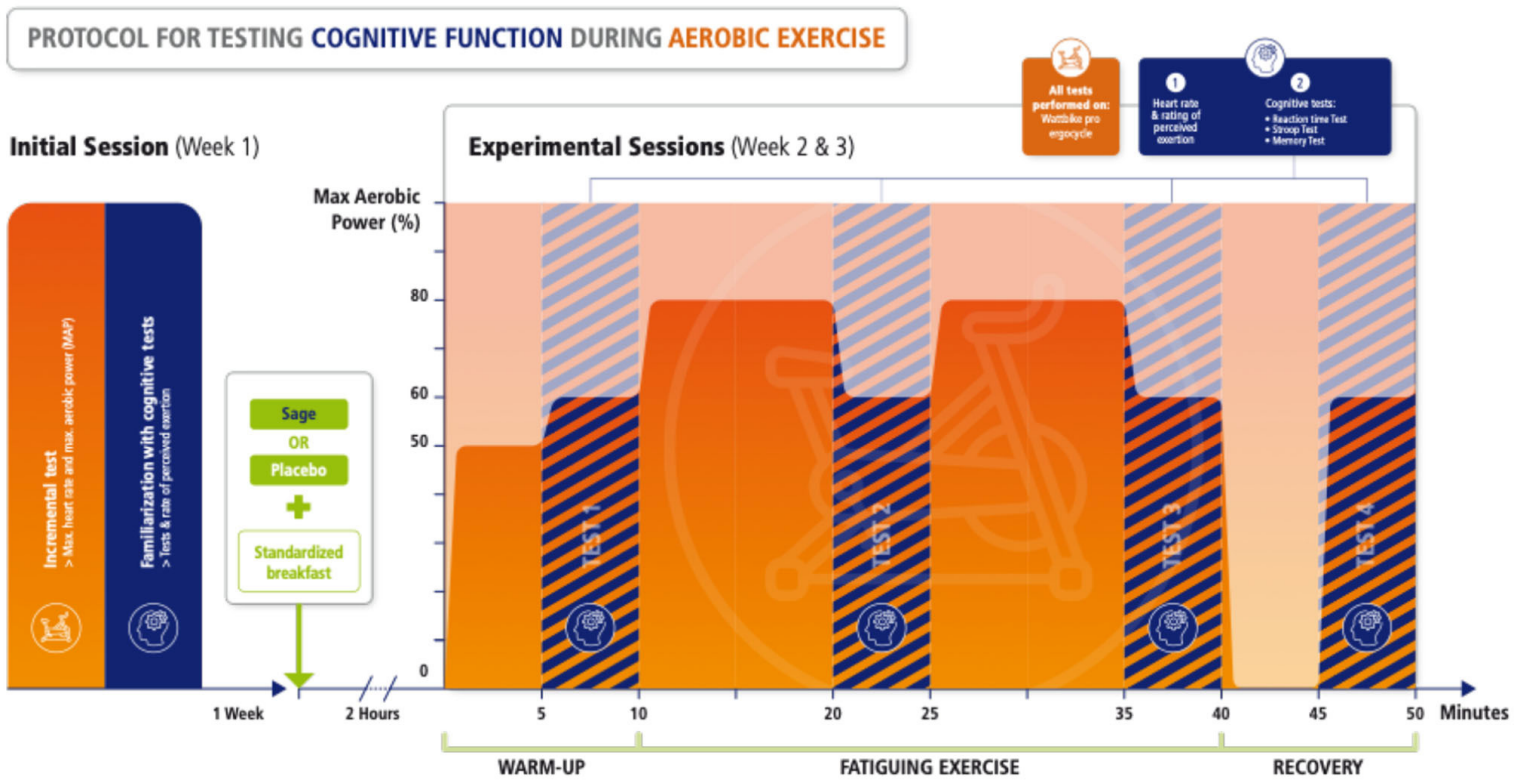
Study flowchart. After a familiarization session (not shown), volunteers performed an initial test session followed by two randomized experimental sessions with SAGE or PLACEBO with 1 week between each session. The experimental sessions were performed on an ergocycle, and cognitive tests were conducted while cycling during the warm-up, during and at the end of the fatiguing exercise and after a short recovery.

**Figure 2:**
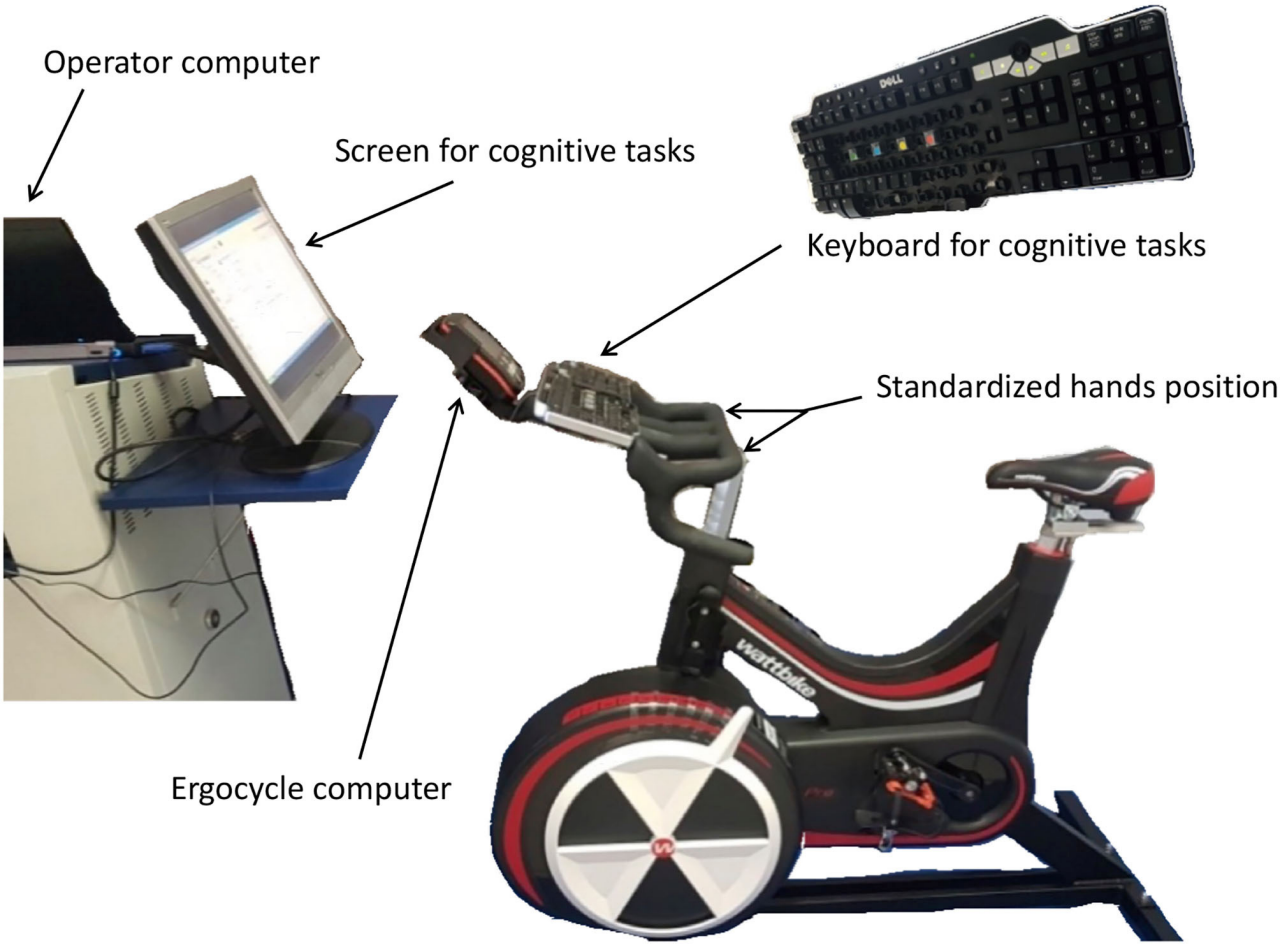
Experimental setup. The experimental procedure was conducted on a Wattbike ergocycle. Cognitive tests were performed with a keyboard positioned on the handlebar and a computer screen facing the ergocycle. During cognitive tests, hands were first positioned on the handlebar. Volunteers then had to press the keyboard as quickly as possible with the index finger of the right hand. For the Stroop test, four colored keys were identified in the middle of the keyboard. For the reaction time, volunteers had to press the red key. For the backward digit span task, volunteers had to write the digits on the numerical keypad.

During the two other experimental sessions, volunteers were tested at different time points during a fatiguing cycling procedure performed 2 h after a supplementation of *Salvia* or placebo (here called SAGE or PLACEBO condition, respectively). SAGE or PLACEBO order was randomly presented by blocks of two using www.randomizer.org website. The fatiguing cycling procedure (Figure 1) consisted in different stages: (i) 5 min of warm-up at 50% of MAP, (ii) first test series (T1), (iii) 10 min at 80% of MAP, (iv) second test series (T2), (v) 10 min at 80% of MAP, (vi) third tests series (T3), (vii) 5-min recovery while pedaling at a very light power output (ranging from 60 to 80 W), and (viii) a final test (T4). Tests (T1–T4) were always performed while cycling for 5 min at 60% of MAP. While cycling, volunteers were requested to remain seated with a pedaling rate ranging between 80 and 90 rpm (except during the 5-min recovery period at very low power output). During the two periods at 80%, volunteers were encouraged at constant time points using standardized sentences. Moreover, care was taken to control power output during the whole procedure in order to produce the same mean power during the two experimental sessions. Three different tests were performed, namely, a Stroop task, a simple reaction time task, and a backward digit span memory task. These tests were selected because they are commonly used to investigate the cognitive function, for instance, in nutrients supplementation studies (15). Moreover, they are often linked to sport-related performance. Stroop and reaction time were selected to investigate attention and vigilance (16). The backward digit span memory task was selected for working memory due to its high cognitive demand (17). Because tests were performed while cycling, a 17-inch computer monitor faced the ergocycle and a keyboard was secured in the front part of the handlebar (Figure 2). Tests were randomly presented but with exactly the same order for a given volunteer. Moreover, heart rate was continuously registered using a Polar heart rate monitor (Polar Electro Oy, Kempele, Finland), and rating of perceived exertion (RPE, 10-point scale) was done at the very beginning of the four test sessions.

### Participants

Twenty-six volunteers (18 men and 8 women) were included in this study. Their mean age ± SD, height, and body mass were 26.1 ± 6.4 years, 173.6 ± 8.4 cm, and 72.3 ± 13.3 kg, respectively. All were physically active with 5.4 ± 1.9 h training per week. Sports were fitness/strength (*n* = 12), running/cycling/swimming (*n* = 12), and team sports (*n* = 4). None reported lower limb injuries within the past 3 months. Prior to participation, they were fully informed about the type of product, expected effects on the cognitive function, purpose of the study, and experimental procedure. However, participants were blinded from our *a priori* hypothesis. All signed an informed consent form. This study was conducted according to the Declaration of Helsinki. Approval was obtained from the University of Burgundy Ethics Committee, and the trial was registered at www.clinicaltrials.gov (NCT04804657). Based on a previous study (18), the sample size was calculated *a priori* using G*Power (version 3.1.9.6, free software available at https://www.psychologie.hhu.de/arbeitsgruppen/allgemeine-psychologie-und-arbeitspsychologie/gpower.html) with a level of significance set at 0.05 and power of 0.9 to detect a large effect (partial-eta-squared: partial η^2^ > 0.14). A sample size of 14 individuals was indicated. The backward digit span memory task was considered as the primary criterion.

### Incremental MAP Test

Participants started cycling at 80 W and 100 W for women and men, respectively. Every 2 min, power was increased using 20 W (women) and 25 W (men) increments. The test was interrupted when participants were unable to maintain the requested cycling rate (80–90 rpm). The last power value maintained at least 60 s was considered as the MAP. During this test, participants were instructed to remain seated all the time and to keep a constant pedaling rate. This power value was used as the reference during the two other experimental testing sessions. Heart rate was continuously measured using a Polar heart rate monitor. The maximal heart rate value was retained.

### Cognitive Tests

An incongruent attentional Stroop task was used (19). It consisted of color words (yellow, blue, red, green) written in a different ink color (either yellow, blue, red, green). Words were presented on a computer screen and appeared centrally on a black background. Volunteers were requested to read the word and to press one of the four colored buttons on a computer keyboard depending on the color of the ink. Volunteers were instructed to press the keyboard as quickly and accurately as they can with the index finger of the right hand. The hand was positioned in a standardized position on the handlebar between trials. Words and ink colors were randomly presented. The test consisted in 32 trials with 1 s between trials. The Stroop accuracy (percentage of correct responses) and averaged Stroop reaction time were measured to monitor cognitive attentional performance. The test was conducted using OpenSesame software (ver 3.3.6 *Lentiform Loewenfeld*, downloadable for free from http://www.cogsci.nl/) (20). Preliminary tests revealed good day-by-day reliability with intraclass correlation coefficients (ICCs), with values being 0.83 and 0.89 for Stroop accuracy and reaction time, respectively.

The simple reaction time test was performed using OpenSesame software. Volunteers were instructed to press a given key on a computer keyboard as quickly as they can with the index finger of the right hand following the visual stimulus. The right hand of the volunteers was positioned on the ergocycle handlebar (standardized position) between each trial. The background color was white, and the stimulus was a draw representing a smiling face. The test consisted in 20 trials with a random period ranging from 1 to 3 s between trials. The averaged reaction time was retained for analyses. Preliminary tests revealed good reliability (ICC = 0.87).

Working memory was evaluated by using the backward digit span memory task [e.g., (3)]. Volunteers had to read a sequence of digits (presented one by one) and then had to write it backward on a computer keyboard. The digit span was presented in an ascending order from three to seven digits. Volunteers had two different and successive trials for each span. A total of 10 spans corresponding to 50 digits were therefore presented. Two scores were measured: digit memory score and span memory score. The digit memory score was the percentage of correct digits (as a function of the total number of digits). The span memory score was the percentage of correct spans (with reference to the 10 spans). This test was conducted using a freely downloadable software (Digit-span-tester.exe ver 2.1.3 available from https://sourceforge.net/projects/digitspantester/). Preliminary tests revealed good reliability with ICC = 0.85 and 0.78 for the digit memory and span memory scores, respectively.

Perception of effort was measured at the very beginning of each test session using a 10-point visual RPE scale (21). Volunteers were asked to rate the conscious sensation of how hard the preceding cycling period was. One corresponded to “very light” exercise and 10 to “very hard” exercise. Simultaneously, the heart rate value was registered in beats per minute (bpm).

### Supplementation

Depending on the randomization, either capsules of 600 mg of *Salvia* extracts or capsules of 600 mg of placebo (maltodextrin) were ingested 2 h before the two experimental sessions. Capsules were of similar appearance. Unfortunately, the taste was impossible to be masked. Some volunteers (*n* = 2) were able to identify the product ingested because of possible reflux. Both the participants and experimenters were blinded from the randomization. Participants were asked to have exactly the same food intake the preceding day. Also, volunteers were asked to have a standardized breakfast 2 h before the experimental sessions. As previously used (13), breakfast was composed of 125 mL orange juice, 80 g wholemeal bread, 20 g of butter, and 20 g of jelly.

The SAGE supplementation was made of Cognivia^TM^ (Nexira, Rouen, France): a mix of *S. officinalis* and *S. lavandulaefolia* (9, 14). It contains 400 mg of aqueous extract from *S. officinalis* leaves characterized for its content in polyphenols (rosmarinic acid, apigenin glucosides, luteolin glucosides, and others) and 200 mg, which contains 50 μL of *S. lavandulaefolia* essential oil characterized for its content in terpenoids (as eucalyptol, camphor, α-, β-pinene, and others) encapsulated with acacia gum. The posology of both active substances has been selected in accordance with descriptions of the most effective dosages described in clinical acute studies introduced above (i.e., 50 μL of *S. lavandulaefolia* essential oil and extract of *S. officinalis* with a ratio equivalent to 2.25 g of dried leaves). Encapsulated powder of essential oil with acacia gum has been proposed to facilitate posology and observance compared to liquid form, and to protect terpenoids from evaporation and oxidation. Moreover, acacia gum has been shown not to compromise the nootropic activities of *S. lavandulaefolia* essential oil (14).

### Statistical Analyses

Statistical analyses were conducted using JASP (version 0.14, JASP Team 2020, University of Amsterdam, available free at https://jasp-stats.org/download/). The normality and sphericity of the data were tested and confirmed by the Shapiro–Wilk and Mauchly's tests. Gender differences for the characteristics of the volunteers and power output during the two experimental sessions were investigated using Student's *t*-test. Then, a two-way (supplementation × time) ANOVA with repeated measures was performed. The supplementation factor corresponded to PLACEBO vs. SAGE. The time factor corresponded to the four test sessions (T1 vs. T2 vs. T3 vs. T4). *Post hoc* tests with Bonferroni corrections were conducted if significant main effects or interactions were present. Partial-eta-squared (partial η^2^) was calculated from ANOVA results, with values of 0.01, 0.06, and above 0.14 representing small, medium, and large differences, respectively (22). Subsequently, qualitative descriptors of standardized effects were used for pairwise comparisons with Cohen's d <0.5, 0.5–1.2, and >1.2 representing small, medium, and large magnitudes of change, respectively (22). *P* < 0.05 was taken as the level of statistical significance for all comparisons. Absolute values are expressed as mean ± SD or mean difference with 95% confidence intervals (95%CI).

## Results

**Figure 3:**
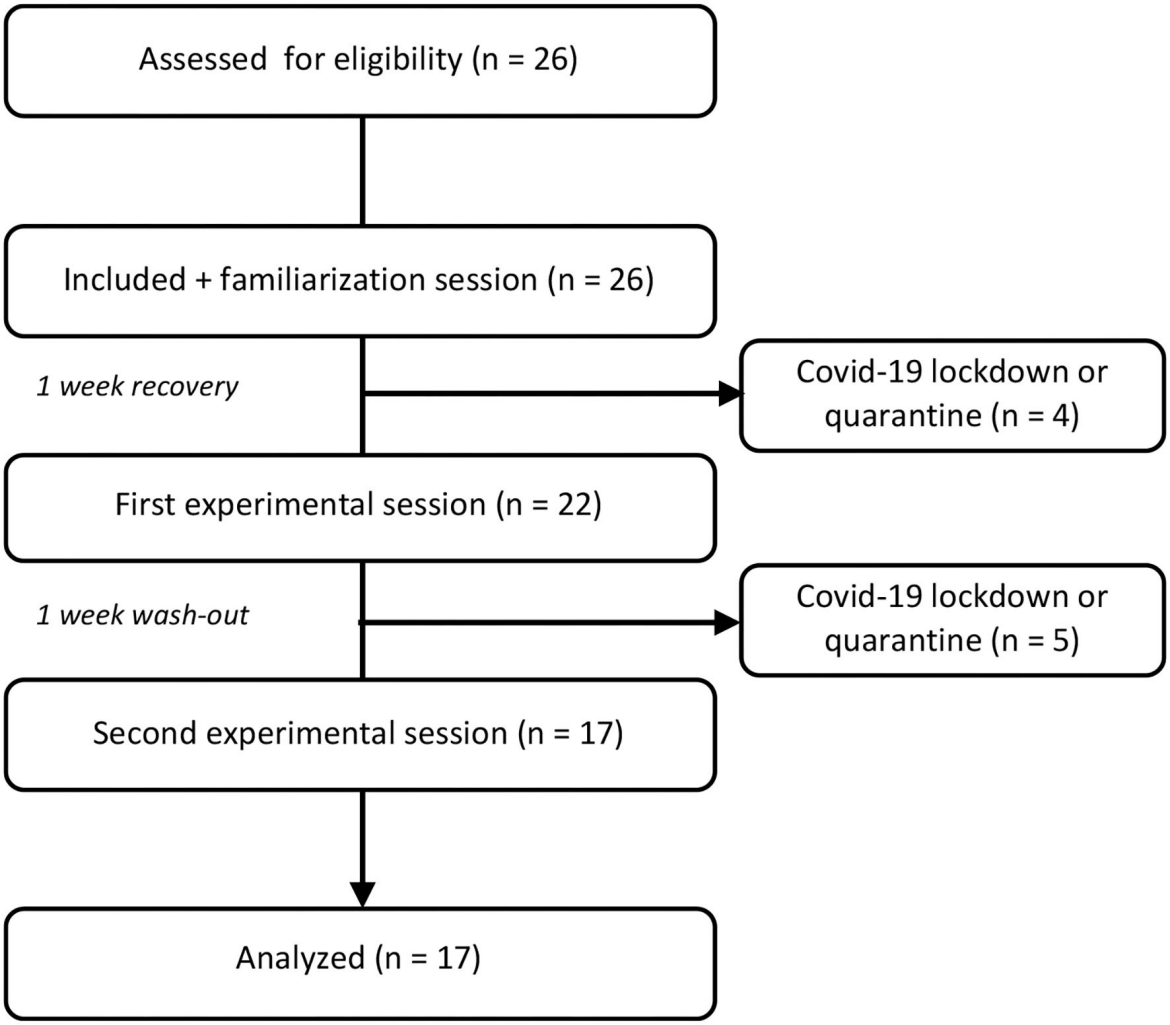
CONSORT flowchart.

Seventeen volunteers were considered for analyses (Table 1). Nine volunteers were lost due to the COVID-19 sanitary crisis (Figure 3). The mean power output during the two experimental procedures (power at 80% MAP) was similar between the two sessions (178.3 ± 50.6 W vs. 179.7 ± 48.8 W during SAGE and PLACEBO, respectively, *P* = 0.196). Results from the two-way ANOVA during the two experimental sessions are presented in Table 2. Briefly, a significant supplementation effect was observed for RPE, reaction time, and responses of the memory test. A significant time effect was obtained for heart rate, RPE, reaction time, and Stroop reaction time. Significant interactions were obtained for RPE and the accuracy of the Stroop test.

**Table 1:** Characteristics of the volunteers analyzed.

**Characteristics**	**Women (*n* = 5)**	**Men (*n* = 12)**	**Women vs. Men**
Age (year)	27.2 ± 7.1 (23.3–31.1)	27.9 ± 7.4 (23.8–32.0)	*p =* 0.944 d = 0.038
Height (cm)^*^	163.8 ± 6.1 (160.4–167.2)	177.1 ± 6.1 173.7–180.5)	*P* <0.001 d = 2.182
Body mass (kg)^*^	59.1 ± 3.5 (57.2–61.0)	77.6 ± 14.2 (69.8–85.4)	*p =* 0.009 d = 1.599
Percentage fat mass^*^	23.0 ± 3.5 (21.1–24.9)	16.4 ± 4.2 (14.1–18.7)	*p =* 0.011 d = 1.551
Weekly training volume (hours per week)	5.6 ± 1.6 (4.7–6.5)	5.3 ± 2.1 (4.1–6.5)	*p =* 0.822 d =0.122
Maximum heart rate (bpm)	188.0 ± 8.5 (183.3–192.7)	193.1 ± 7.6 (188.9–197.3)	*p =* 0.262 d = 0.620
Maximal aerobic power (W)	191.0 ± 55.8 (160.3–221.7)	250.0 ± 64.3 (214.6–285.4)	*p =* 0.143 d = 0.823

*Mean ± SD (95%CI). Gender differences are presented with p-values and Cohen's d*.

* *p < 0.05*.

**Table 2:** Results from the two-way ANOVA during the two experimental sessions.

**Parameter**	**Effect**	**F**	***p***	**Partial **η^2^****
Heart rate	Supplementation	0.160	0.695	0.01
	Time	105.6	<0.001^*^	0.868
	Supplementation × Time	2.722	0.055	0.145
RPE	Supplementation	12.821	0.002^*^	0.445
	Time	239.5	<0.001^*^	0.937
	Supplementation × Time	5.521	0.002^*^	0.257
Reaction time	Supplementation	6.290	0.023^*^	0.282
	Time	2.955	0.042^*^	0.156
	Supplementation × Time	1.157	0.336	0.067
Stroop reaction time	Supplementation	0.005	0.944	0
	Time	5.057	0.004^*^	0.240
	Supplementation × Time	0.355	0.786	0.022
Stroop accuracy	Supplementation	1.758	0.203	0.099
	Time	0.024	0.995	0.001
	Supplementation × Time	3.845	0.015^*^	0.194
Digit memory score	Supplementation	3.657	0.074	0.186
	Time	2.743	0.053	0.146
	Supplementation × Time	1.772	0.165	0.100
Span memory score	Supplementation	4.781	0.044^*^	0.230
	Time	1.710	0.177	0.097
	Supplementation × Time	0.824	0.487	0.049

* *p < 0.05*.

**Figure 4:**
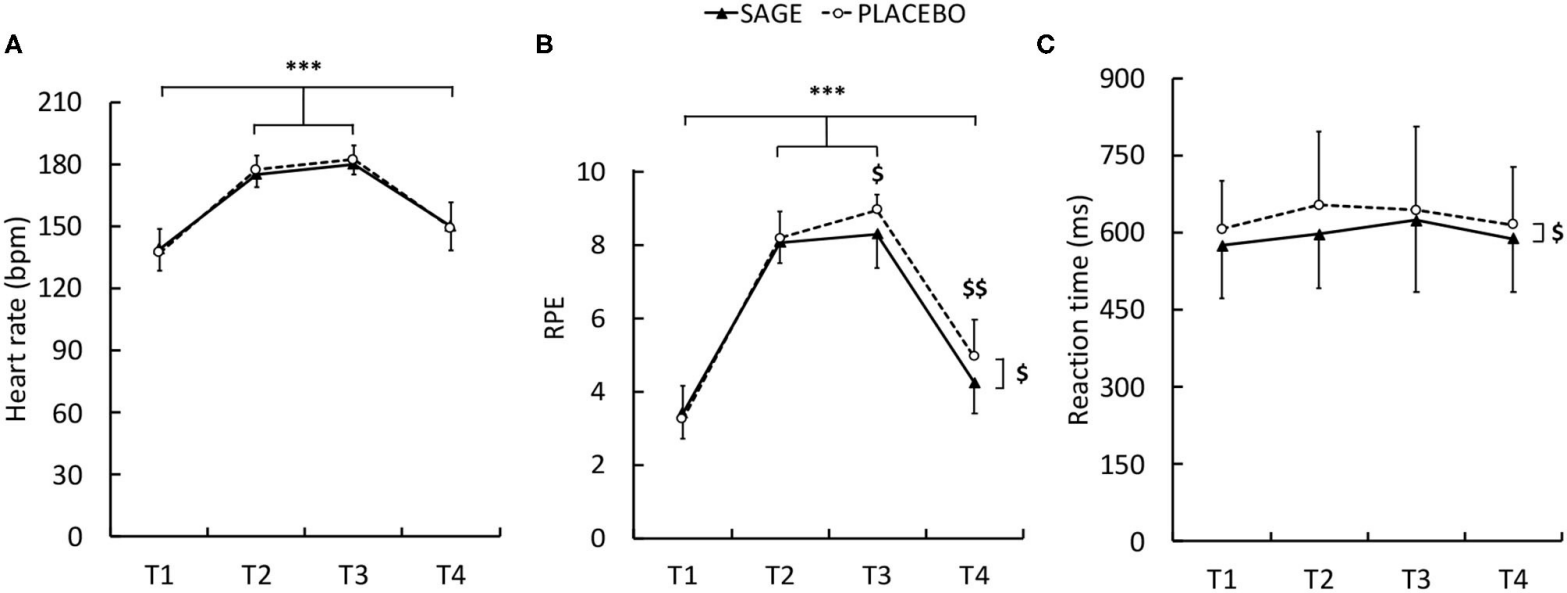
Heart rate **(A)**, RPE [rate of perceived exertion, **(B)**], and reaction time **(C)** with SAGE and PLACEBO supplementations during the different tests. T1, T2, T3, and T4 were tests following warm-up, first and second fatiguing stages, and recovery, respectively. *** significant difference between tests whatever the supplementation (*P* < 0.001). $ significant difference between the supplementation for a given time point (RPE) or main effect (reaction time) (*P* < 0.05).

*Post hoc* analyses revealed that heart rate was significantly greater during T2 and T3 (during the fatiguing exercise) as compared to T1 and T4 (warm-up and recovery, respectively) (Figure 4, *P* < 0.001). No difference was observed between T2 and T3 (mean difference ± SD (95%CI): 4.9 ± 11.7 bpm (−2.9; 12.7), d = 0.419, small, *P* = 0.545). Heart rate remained significantly greater at T4 than at T1 (mean difference ± SD (95%CI): 11.5 ± 11.7 bpm (3.7; 19.4), d = 0.985, medium, *P* = 0.001). The *post hoc* analysis conducted on the interaction for RPE revealed various effects. With SAGE and PLACEBO, RPE was significantly lower at T1 as compared to T2, T3, and T4 (Figure 4, *P* < 0.001). For both SAGE and PLACEBO, RPE was significantly greater at T2 and T3 than at T4 (*P* < 0.001). However, RPE was significantly greater with PLACEBO as compared to SAGE at T3 [mean difference ± SD (95%CI): 0.6 ± 0.7 (0.1; 1.2), d = 0.901, medium, *P* = 0.005] and T4 [mean difference ± SD (95%CI): 0.7 ± 0.7 (0.1; 1.3), d = 0.705, medium, *P* = 0.002]. The main supplementation effect revealed lower RPE, all time points merged, with SAGE as compared to PLACEBO [mean difference ± SD (95%CI): 0.3 ± 0.3 (0.1; 0.5), d = 0.868, medium, *P* = 0.002].

**Figure 5:**
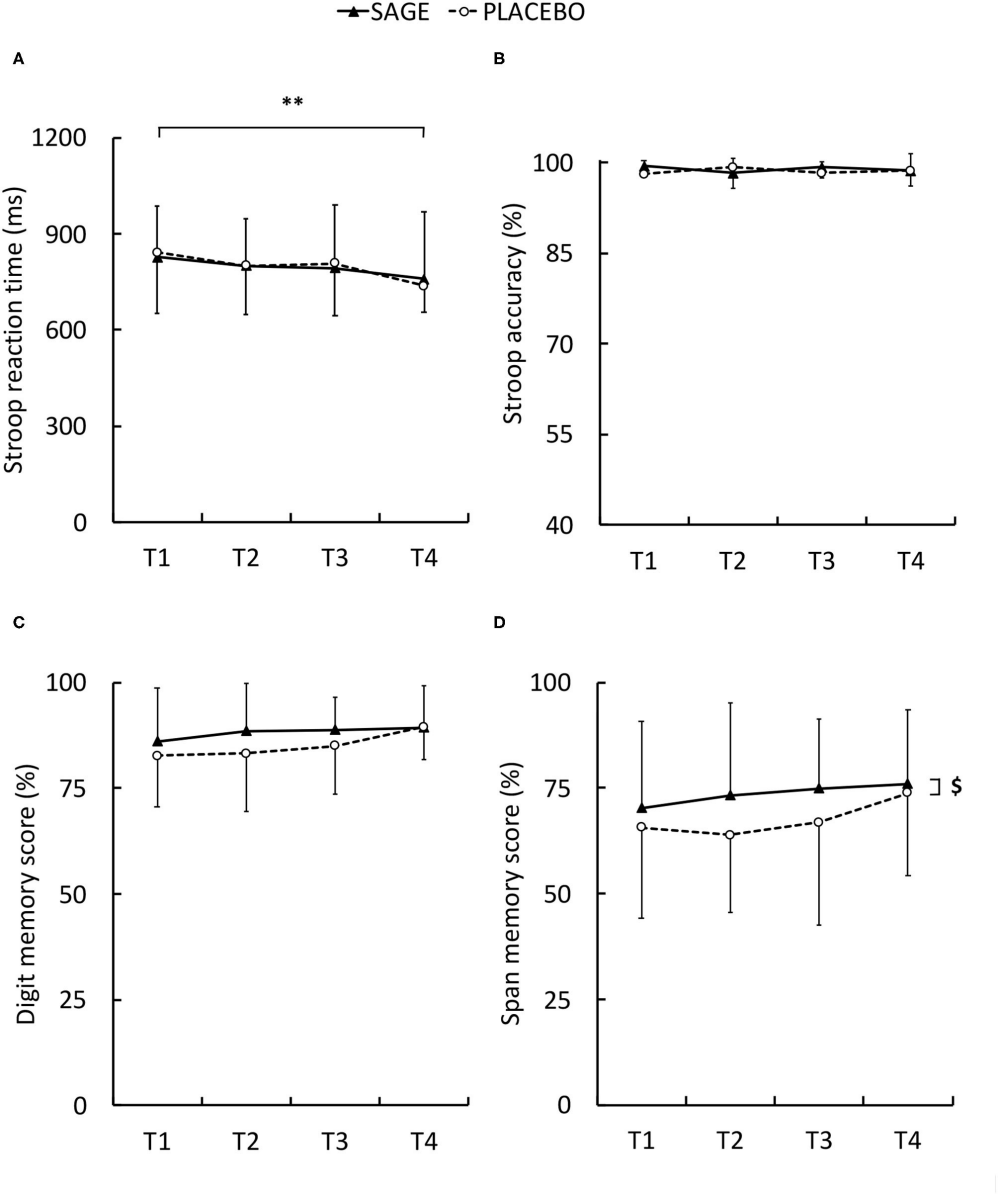
Stroop reaction time **(A)**, Stroop accuracy **(B)**, digit memory score **(C)**, and span memory score **(D)** with SAGE and PLACEBO supplementations during the different tests. T1, T2, T3, and T4 were tests following warm-up, first and second fatiguing stages, and recovery, respectively. ** significant difference between tests whatever the supplementation (*P* < 0.01). $ main significant effect between the supplementation (*P* < 0.05).

Reaction time was not altered during the exercise. However, reaction time (Figure 4) was significantly longer with PLACEBO as compared to SAGE [mean difference ± SD (95%CI): 33.7 ± 55.4 ms (5.2; 62.2), d = 0.608, medium, *P* = 0.023]. During the Stroop task, reaction time (Figure 5) was significantly longer at T1 as compared to T4 [mean difference ± SD (95%CI): −86.4 ± 92.4 ms (−24.7;148.1), d = 0.935, medium, *P* = 0.02]. The *post hoc* analysis did not reveal any differences for Stroop accuracy. During the digit span memory test, the results revealed a significant supplementation effect (Figure 5). A significant greater span score was measured with SAGE as compared to PLACEBO [mean difference ± SD (95%CI): 6.0% ± 11.4 (0.2; 11.9), d = 0.53, medium, *P* = 0.044].

## Discussion

This study aimed to explore the acute effects of a supplementation of *S. officinalis* and *S. lavandulaefolia* on cognitive functions in athletes performing a fatiguing cycling task. The main findings are as follows: (1) The combination of *Salvia* improved the cognitive function (as witnessed by perceived exertion, working memory, and reaction time) throughout the experimental procedure and (2) the positive effects were maintained with fatigue. The present findings partly confirmed our initial hypothesis. We can conclude that an acute supplementation with a combination of *S. officinalis* and *S. lavandulaefolia* is beneficial on some cognitive function outcomes in the context of an endurance cycling exercise.

The results of this study confirmed previously published observations that revealed beneficial effects of *Salvia* on the cognitive function (9–11). These positive results were previously obtained with chronic supplementation in humans suffering from different diseases such as Alzheimer's disease or in patients with chronic fatigue syndrome (23, 24). Acute *Salvia* supplementation was also beneficial in healthy individuals for mood, memory, or attention (7, 9–11, 25). More specifically, in healthy individuals, the two species of *Salvia* under investigation in the present study (*S. officinalis* and *S. lavandulaefolia*) have, separately, demonstrated improvements for immediate or delayed word recall, speed of memory, and Stroop test (7, 10, 26). These positive effects have been observed within the first 4 h post-ingestion (6, 26). The combination of these two species has been explored more recently (9). Similar positive effects, for instance in working memory, have been obtained in healthy individuals just 2 h after supplementation but also after a chronic administration (9, 14).

The nootropics properties of *Salvia* are partly described and begin to be explained (5). Inhibition of the acetylcholinesterase is often proposed as a mechanism for memory retention (27, 28). Interestingly, acetylcholinesterase inhibition is demonstrated for the two species of *Salvia* tested here whether it is an aqueous extract from leaves or essential oil. In addition, terpenes particularly identified in *S. lavandulaefolia* essential oil are able to attenuate oxidative stress by inhibiting reactive oxygen species production (8). Other effects originating from the phenolic constituents of *Salvia* include antioxidant, anti-inflammatory activities in neurons as well as increases in brain-derived neurotrophic factor (5). A preclinical trial also demonstrated that chronic ingestion of *S. officinalis* combined with *S. lavandulaefolia* increased the expression of calcium/calmodulin-dependent protein kinase II (14). This enzyme has large impact for improving working memory and cognitive processes since it acts in numerous neuronal functions, including neurotransmitter metabolism, neuronal signal transduction, or synaptic plasticity [see (29, 30)]. However, further studies are clearly needed to verify these hypotheses after a single intake and elucidate the *Salvia*-induced mechanisms.

To date and to the best of our knowledge, no study has explored the acute effects of *Salvia* in the context of sport, physical activity, or exercise-induced fatigue. In humans, fatigue has previously been considered in those complaining of chronic fatigue (23) with chronic supplementation of *Salvia miltiorrhiza* combined with *Astragali*. In healthy individuals, fatigue from exercise was explored with chronic supplementation in a single study performing a downhill running with *S. miltiorrhiza* combined with *panax ginseng* (31). A faster recovery of the joint range of motion and a lower increase in creatine kinase were obtained with supplementation after this eccentric-type exercise (31). Moreover, interleukin-6 increased with the placebo group, while it remained unchanged in the supplementation condition (31). Two other studies used animals performing a forced swimming test with chronic supplementation of Tanshinone IIA, a constituent from *S. miltiorrhiza* (32), or *S. miltiorrhiza* combined with *Astragali* (33). These two studies revealed an anti-fatigue effect of *Salvia* with reductions in serum glucose and lactate levels and by attenuating oxidative and inflammatory response (32, 33). Taken together, these studies with chronic supplementation confirmed that *Salvia* could be beneficial during exercise.

This study was the first to explore the acute effects of *Salvia* supplementation during exercise. Despite positive findings in some outcomes, our results are partly in contradiction with our initial hypothesis. Indeed, we hypothesized that the positive effects of the supplementation would be exacerbated with fatigue. Considering cognitive tests, no additional supplementation effect was obtained with fatigue. It signified that the positive effects of the supplementation were obtained in fresh conditions and were maintained during the exercise. In contrast, RPE was significantly altered during the intensive cycling exercise and during recovery (lower values with SAGE as compared to PLACEBO). At first sight, this outcome could appear trivial in sporting situations, but it is of paramount importance. Indeed, the central nervous system is well-known to play a very important role in the fatigue process (34, 35). For instance, numerous studies have demonstrated the negative impact of mental fatigue in sport-specific situations such as decision making in team sports or reaction time (35–38). After a demanding cognitive task, RPE was one of the most altered outcomes (36, 38, 39). The authors concluded that mental fatigue would limit exercise tolerance through higher perception of effort (39) and often consider RPE as a limiting factor for endurance performance instead of a simple marker of exercise intensity (40). Considering this psychobiological approach, the lower RPE observed with *Salvia* clearly suggested that supplementation could positively impact performance during fatiguing exercises. Such hypothesis should be further investigated since the present procedure was not conducted until complete exhaustion.

The lower RPE obtained with *Salvia* supplementation is not surprising. *Salvia* is a well-described supplement with numerous active constituents, including polyphenols with antioxidant effects (5, 12). Phenolic constituents are rosmarinic acid, caffeic acid, salvianolic acid, sagecoumarin, and sagerinic acid (5, 9). Polyphenols have been demonstrated to positively impact endurance performance in recreational and elite athletes (13, 41) concomitantly with a delay in maximal perceived exertion. Interestingly, one species of *Salvia* considered here (*S. officinalis*) has the highest antioxidant effects (42). Furthermore, the monoterpenes of the co-administered *S. lavandulaefolia* have been demonstrated to inhibit reactive oxygen species production (8). One should acknowledge that other studies failed to identify positive effects of polyphenols on endurance performance (43, 44). These conflicting findings could be related to the training status. Elite athletes (43) or well-training cyclists (44) were included, while physically active individuals were tested here.

Taken together, our results demonstrated the positive effects of *Salvia* during fatiguing exercises with lower RPE and maintained cognitive performance. Such finding has large impact for performance but also for injury prevention. Indeed, a recent study pointed out altered kinematics of landing that could increase injury risks in people conducting a perceptual-cognitive task combined with fatigue (45) such as those regularly occurring in numerous sports. Increasing awareness with, for example, cognitive training has been suggested to be efficient as prevention programs for a better neuromuscular control (46). Combining these preventive programs with supplementation could be hypothesized to emphasize prevention. Therefore, investigating the effects of supplementation in specific technical tasks would be of interest to confirm a potential positive effect for injury prevention.

Several limitations could be acknowledged. First, like in other studies, although the present study was double-blind, some volunteers were able to identify the product ingested. It is attributed to the herbal taste during some reflux at the end of the fatiguing exercise. Nevertheless, statistical analyses were similar when including or excluding these volunteers (*n* = 2). In addition, we decided to evaluate the cognitive function while cycling. This double task, often observed during sporting events, could be a limitation. Nevertheless, care was taken to familiarize individuals during two separate sessions. The fatiguing exercise consisted in two 10-min high-intensity cycling (with RPE > 8). This exercise was designed to be achievable by physically active individuals; it could be interesting to repeat such experimental design with a greater exercise difficulty, for instance, as often performed, until exhaustion (13). Finally, cognitive function was investigated using some specific tasks. It should be acknowledged that the Stroop task was not sensitive to fatigue with values very close to the maximal performance (100% of accuracy). Additional measurements for the Stroop test could be suggested to increase its sensitivity to fatigue or to the supplementation. Additional cognitive tasks could be applied in order to have a more exhaustive view of *Salvia* acute effects. Moreover, additional cognitive tasks, specific to some real-world sporting situations (for example, reaction time while playing tennis), should be tested in order to conclude that the observed effects could be translated to sporting events. In line with this perspective, the exercise under investigation should also be more specific (for example, while modeling a sporting event).

## Conclusions

In conclusion, this study is consistent with previous experiments conducted in healthy individuals after acute *Salvia* supplementation. As compared to placebo, it revealed improved cognitive function (perceived exertion, reaction time, and working memory) after an acute *Salvia* intake (2 h after ingestion). Most effects were observed independently of the time point (before, during, or after a fatiguing cycling exercise). It is therefore suggested that *Salvia* effects are not negatively or positively affected by the exercise performed. *Salvia* demonstrated universal effects in fresh or fatigued conditions. It has a potential impact in the context of sport performance and more generally in the context of physical activity but requires more real-world exercises and cognitive tasks.

## Data Availability Statement

The raw data supporting the conclusions of this article will be made available by the authors, without undue reservation.

## Ethics Statement

The studies involving human participants were reviewed and approved by University of Burgundy Institutional Ethics Committee. The patients/participants provided their written informed consent to participate in this study.

## Author Contributions

NB conceived and designed the experiment, analyzed the data, performed statistical analysis, and wrote the first draft of the paper. AN, NA, and CC performed the experiments. AN and CC performed data collection. NB and DG interpreted the data. All authors reviewed the manuscript and approved the final version of the paper.

## Funding

This study was supported by the Centre d'Expertise de la Performance from the Université of Bourgogne. The product under investigation was provided by Nexira. The authors declare that this study received funding from Nexira to cover the costs of publication. The funder was not involved in the study design, collection, analysis, interpretation of data, the writing of this article or the decision to submit it for publication.

## Conflict of Interest

DG was employed by Nexira. The remaining authors declare that the research was conducted in the absence of any commercial or financial relationships that could be construed as a potential conflict of interest.

## Publisher's Note

All claims expressed in this article are solely those of the authors and do not necessarily represent those of their affiliated organizations, or those of the publisher, the editors and the reviewers. Any product that may be evaluated in this article, or claim that may be made by its manufacturer, is not guaranteed or endorsed by the publisher.

## Abbreviations

95%CI: 95% confidence intervals
ANOVA: analysis of variance
bpm: beats per minute
MAP: maximal aerobic power
partial η^2^: partial-eta-squared
RPE: rating of perceived exertion
rpm: revolutions per minute
SD: standard deviation
T1, T2, T3, and T4, tests during warm-up, during the fatiguing exercise, at the end of the fatiguing exercise, and after recovery: respectively.
